# Nonalcoholic fatty liver disease: an emerging threat to obese and diabetic individuals

**DOI:** 10.1111/nyas.12016

**Published:** 2013-01-30

**Authors:** Howard C Masuoka, Naga Chalasani

**Affiliations:** Division of Gastroenterology and Hepatology, Department of Medicine, Indiana University School of MedicineIndianapolis, Indiana

**Keywords:** steatosis, steatohepatitis, fatty liver, thiazolidinediones

## Abstract

Nonalcoholic fatty liver disease (NAFLD) is the most common liver disease in the Western world and its incidence is increasing rapidly. NAFLD is a spectrum ranging from simple steatosis, which is relatively benign hepatically, to nonalcoholic steatohepatitis (NASH), which can progress to cirrhosis. Obesity, insulin resistance, type 2 diabetes mellitus, and dyslipidemia are the most important risk factors for NAFLD. Due to heavy enrichment with metabolic risk factors, individuals with NAFLD are at significantly higher risk for cardiovascular disease. Individuals with NAFLD have higher incidence of type 2 diabetes. The diagnosis of NAFLD requires imaging evidence of hepatic steatosis in the absence of competing etiologies including significant alcohol consumption. Liver biopsy remains the gold standard for diagnosing NASH and for determining prognosis. Weight loss remains a cornerstone of treatment. Weight loss of ∼5% is believed to improve steatosis, whereas ∼10% weight loss is necessary to improve steatohepatitis. A number of pharmacologic therapies have been investigated to treat NASH, and agents such as vitamin E and thiazolidinediones have shown promise in select patient subgroups.

## Introduction

Nonalcoholic fatty liver disease (NAFLD) is the name given to a spectrum of liver disorders associated with hepatic steatosis that is not due to significant alcohol consumption or other secondary causes, such as steatogenic medication, or inborn errors of metabolism (Table 1). This disorder encompasses a wide range of diseases, from simple steatosis, which is relatively benign, to hepatic inflammation, hepatocyte injury, and fibrosis, a syndrome referred to as nonalcoholic steatohepatitis (NASH), which can progress to cirrhosis.^1^–^3^ Over the last three decades, NAFLD has emerged as one of the leading causes of cirrhosis in the United States, and a large proportion of individuals who previously had been classified as having cryptogenic cirrhosis are now believed to have cirrhosis due to NASH.^4^

**Table 1:** Common causes of hepatic macrovesicular steatosis

- Obesity, type 2 diabetes, and dyslipidemia (NAFLD)
- Excessive alcohol consumption
- Hepatitis C (genotype 3)
- Wilson's disease
- Lipodystrophy
- Starvation
- Parenteral nutrition
- Abetalipoproteinemia and hypobetalipoproteinemia
- Medications (e.g., amiodarone, methotrexate, tamoxifen, corticosteroids)

NAFLD is becoming an increasingly important health issue. In Western nations, including the United States, NAFLD has become the most common cause of chronic liver disease. The rate of NAFLD is increasing likely due to the rising prevalence of associated conditions such as obesity and type 2 diabetes mellitus (T2DM). It has been projected that, within the next two decades, NASH will become the predominant cause of cirrhosis requiring orthotopic liver transplantation.^5^,^6^

This review focuses on clinical aspects of NAFLD, such as epidemiology, natural history, need for liver biopsy, and treatment options. A detailed discussion of pathogenesis, genetic investigations, and histological classification is beyond the scope of this review, and interested readers are referred to several recent excellent reviews.^7^–^14^ A recently published multi-society practice guideline offers guidance on the diagnosis and management of NAFLD for practicing healthcare providers.^15^

## Epidemiology of NAFLD

The epidemiology and natural history of NAFLD remain incompletely understood, although studies have begun to more clearly elucidate them. The incidence of fatty liver disease has been examined in only a few studies. In an early study from Japan, the incidence of NAFLD was estimated to be 31 per 1,000 person-years based on the incidence of elevated aminotransferases as a surrogate for NAFLD.^16^ In a more recent Japanese study, which employed biennially abdominal ultrasound examination of 1,635 Nagasaki atomic bomb survivors without NAFLD at baseline, the incidence of NAFLD was 19.9 per 1000 person-years (22.3 for men, 18.6 for women) and peaked in the sixth decade of life.^17^ The annual incidence of NAFLD in England was estimated to be 29 per 100,000 individuals in one study, but this frequency is likely an underestimation because it was based on outpatient hepatology referrals.^18^ In this study, the incidence of NAFLD was much higher than other types of chronic liver disease.

The estimated prevalence of NAFLD varies widely likely secondary to difference in the population studied and the method used to detect NAFLD. The prevalence of suspected NAFLD based on elevated aminotransferases without imaging is between 7% and 11%, but this likely is an underestimation because aminotransferases can be normal in individuals with NAFLD.^19^ The prevalence of significant hepatic steatosis in potential living donors for liver transplantation was 20% by liver biopsy.^20^ The prevalence of NAFLD in participants of the Dallas Heart Study, a multi-ethnic population based study in Dallas County, Texas, was 34% using magnetic resonance spectroscopy for hepatic triglyceride quantitation.^21^ The estimated prevalence of NASH is lower, ranging from 3 to 5% of the general population whereas the prevalence of NASH-related cirrhosis is unknown.^19^

Ethnicity has a significant impact on the prevalence of NAFLD. In the Dallas Heart Study, the prevalence of hepatic steatosis was 45% in Hispanics, 33% in non-Hispanic Caucasians, and 24% in African Americans.^21^ This difference in prevalence was only partially explained by differences in obesity and insulin resistance especially in African Americans where the prevalence of NAFLD was lower than in Caucasians with similar risk factors. This condition is highly prevalent in Asian population as well. For example, in a Korean study of potential liver donors who underwent liver biopsy, the presence of NAFLD was 51%, with 10% revealing >30% steatosis and 2.2% with NASH.^22^

Gender has a significant impact on the prevalence of NAFLD, with most epidemiologic studies demonstrating almost twice the prevalence of NAFLD in males compared with females. In a study of 26,527 subjects undergoing medical checkups in China, the prevalence of NAFLD by abdominal ultrasound was 31% in men and 16% in women.^23^ Similarly, a population based study in India demonstrated a 25% prevalence of NAFLD in men compared with 14% in women.^24^ The prevalence of NAFLD in the Dallas Heart Study was 42% in white men compared with only 24% in white women and this difference was not attributable to differences in body weight or insulin sensitivity.^21^ However, this study found no gender difference in the prevalence of NAFLD in Hispanic and Black Americans. Studies have suggested that estrogen may reduce the risk of developing NAFLD.^25^

Jejunoileal bypass surgery has long been recognized as a cause of NAFLD likely due to the rapidity of weight loss and bacterial overgrowth leading to increased levels of endotoxin in the portal circulation.^26^,^27^ This procedure has now been abandoned, and the current foregut bariatric surgical procedures are not believed to cause or significantly worsen NAFLD. The efficacy and role of foregut bariatric surgery in individuals with NAFLD will be discussed later in detail. Pancreaticoduodenectomy has also been associated with an increased risk of subsequent development of NAFLD. In one series from Japan, the incidence of NAFLD after pancreatico-duodenectomy NAFLD was 37% with 10% exhibiting NASH.^28^

A number of studies have examined the prevalence of NAFLD in patients attending obesity clinics or undergoing bariatric surgery. The reported prevalence of NAFLD in this group ranges from 57% to 91% whereas the prevalence of NASH ranged from 26% to 37%.^22^,^29^–^31^ Unsuspected cirrhosis was 1.6–1.7%.^29^,^30^

Although obesity, insulin resistance, T2DM, and dyslipidemia are the most important risk factors, other endocrine conditions, such as hypothyroidism, hypopituitarism, hypogonadism, and polycystic ovary syndrome, are also associated with NAFLD (Table 2).^32^–^35^

**Table 2:** Risk factors associated with NAFLD

Major risk factors	Conditions with emerging association
Truncal obesity and insulin resistance	Hypothyroidism
	Obstructive sleep apnea
Type 2 diabetes mellitus	Hypopituitarism
	Hypogonadism
Hypertriglyceridemia	Pancreaticoduodenal
Metabolic syndrome	resection
	Polycystic ovary syndrome

## Relationship of NAFLD to obesity and diabetes

The majority of patients with NAFLD have metabolic risk factors, such as obesity, T2DM, and dyslipidemia. Conversely, the presence of NAFLD is a risk factor for the subsequent development of some metabolic disorders such as T2DM. T2DM is not only a risk factor for the development of NAFLD but also a risk factor for the development of cirrhosis and hepatocellular carcinoma.^36^,^37^

Obesity and dyslipidemia are well-established risk factors for NAFLD. A Japanese study found that obesity, low high-density lipoprotein-cholesterol, hypertriglyceridemia, glucose intolerance, and hypertension were risk factors for the development of NAFLD, though in the multivariate analysis, only obesity, hypertriglyceridemia, and hypertension remained predictive.^17^ Similarly, a Korean study of living donors found obesity, older age, and hypertriglyceridemia were independent risk factors for NAFLD.^22^ The prevalence of NAFLD in patients referred to a lipid clinic was found to be 50% in one series.^38^ Visceral fat accumulation appears to be a significant risk for the development of NAFLD. A study from Japan found that the severity of hepatic steatosis by ultrasound was positively correlated with visceral fat accumulation and insulin resistance in both obese and nonobese subjects, suggesting that hepatic steatosis may be influenced by visceral fat accumulation regardless of body mass index.^39^

Metabolic syndrome, as defined by the Adult Treatment Panel (ATP) III criteria, is defined by the presence of three or more of the following: (1) waist circumference greater than 102 cm in men or greater than 88 cm in women; (2) triglyceride level greater than 150 mg/dL (1.7 mmol/L) or drug treatment for elevated triglycerides; (3) high-density lipoprotein (HDL) cholesterol level less than 40 mg/dL (1.03 mmol/L) in men and less than 50 mg/dL (1.29 mmol/L) in women or on drug treatment for low HDL; (4) systolic blood pressure ≥130 mm Hg or diastolic pressure ≥85 mm Hg or treatment for hypertension; and (5) fasting plasma glucose level ≥110 mg/dL or drug treatment for elevated blood glucose.^40^ Patients with metabolic syndrome have an increased prevalence of NAFLD with 86% of patients with metabolic syndrome having NAFLD, 24% exhibiting steatohepatitis, and unexpected cirrhosis in 2% by liver biopsy.^41^ An observational study from Japan demonstrated that men and women with metabolic syndrome at baseline were more likely to develop NAFLD during a 14 month follow-up with an adjusted odds ratio of 4.0 and 11.2, respectively.^42^ Conversely, NAFLD increases the risk for subsequent development of metabolic syndrome.^43^

Insulin resistance and diabetes are both very important risk factors for the development of NAFLD. Several studies have demonstrated that an elevated insulin resistance index, either HOMA-IR greater than 5.8 or FAIR score of 2 or greater, is a risk factor for the development of NAFLD in overweight nondiabetic individuals.^29^,^30^ In one series of patients undergoing gastric bypass, the odds of NASH were 128 times greater and the odds of severe fibrosis 75 times greater in patients with T2DM than in those without T2DM.^31^ Several recent studies have observed that adipose tissue insulin resistance (Adipo IR) may be an important predictor of liver histology in individuals with NAFLD and may indeed predict fibrosis progression.^44^,^45^ There are unconfirmed reports that T1DM is associated with NAFLD but these reports have not provided histological confirmation of hepatic fat accumulation.^46^,^47^

NAFLD is an independent risk factor for the development of T2DM. A study from South Korea examined the effect of NAFLD on the risk of subsequent development of T2DM in patients with impaired fasting glucose. The incidence of T2DM in the NAFLD group was 9.9% compared with 3.7% in the non-NAFLD group suggesting that NAFLD has an independent and additive effect on the development of T2DM in individuals with insulin resistance.^48^

## Natural history of NAFLD

The natural history of NAFLD has been examined in a number of studies, although it remains incompletely understood. The majority of patients with simple steatosis will not develop NASH or advanced fibrosis. However, simple steatosis may not be totally benign as a portion of patients will progress to NASH. Approximately 23% of patients with simple steatosis noted on an initial liver biopsy were found to have NASH on a subsequent liver biopsy 36 months later.^37^ However, patients with NASH are certainly at risk for histologic progression including the development of cirrhosis. Age and the degree of inflammation observed in the initial liver biopsy are risk factors for progression to advanced fibrosis.^49^ Although only a small percentage of patients with NAFLD will eventually develop cirrhosis, given the large number of patients with NAFLD, this represents a significant disease burden. It is estimated that approximately 5% of patients with NAFLD develop cirrhosis.^1^

Subjects with NAFLD are at significantly higher risk for cardiovascular disease, and in fact cardiovascular events are their most common cause of death (Table 3). This association likely reflects high prevalence of metabolic risk factors in individuals with NAFLD, rather than additional atherogenic risk posed by the hepatic steatosis itself. Compared to simple steatosis, NASH has higher liver-related mortality with an odds ratio (OR) of 5.71 for NASH and an OR of 10.06 for NASH with advanced fibrosis.^50^ The cardiovascular mortality was found to be similar between patients with simple steatosis and those with NASH.^50^ When compared to patients with chronic hepatitis C, patients with NAFLD with advanced fibrosis or cirrhosis have lower rates of liver-related complications and hepatocellular cancer but similar overall mortality.^51^

**Table 3:** Incidence of cardiovascular disease in patients with NAFLD in selected longitudinal studies^148^

Author (Ref.)	Number of subjects	Diagnosis of NAFLD	Follow-up duration (years)	Proportion of deaths due to cardiovascular disease	Comment
Soderberg^145^	118	Histology	24 (median)	30%	CVD is the most common cause of death
Ekstedt^2^	129	Histology	13.7 ± 1.3 (mean)	16%	CVD is the most common cause of death
Adams^1^	421	Imaging	7.6 ± 4.0 (mean)	25%	CVD is the 2nd most common cause of death after malignancy
Dam-Larsen^146^	170	Histology	20.4 (median)	38%	CVD is the most common cause of death
Rafiq^147^	173	Histology	18.5 (median)	12.7%	CVD is the most common cause of death

Reproduced with permission.

## Diagnosis of NAFLD

Diagnosis of suspected NAFLD involves establishing that the patient satisfies the diagnostic criteria for NAFLD and excluding the presence of other coexisting liver diseases. Diagnosis of NAFLD requires demonstration of hepatic steatosis by imaging or histology and exclusion of significant alcohol use or other secondary causes of steatosis. In addition to alcohol consumption, secondary causes of hepatic steatosis include medications, chronic hepatitis C infection, parenteral nutrition, and Wilson's disease (Table 1).

### Liver biochemistries and other laboratory testing

In patients with suspected NAFLD, initial laboratory evaluation typically involves obtaining liver biochemistries and exclusion of chronic viral hepatitis, hemochromatosis, Wilson's disease, and autoimmune hepatitis via appropriate diagnostic tests.

In patients with NAFLD, serum aminotransferases can be normal or mildly elevated. Serum aminotransferases generally wax and wane and they rarely exceed 200 U/L. Typically, ALT exceeds AST although the serum AST frequently exceeds ALT when there is advanced fibrosis.^52^ Alkaline phosphatase can also be elevated as well, and patients can have only an isolated elevation of serum alkaline phosphatase with normal aminotransferase levels.^53^ NAFLD is the most common cause of incidentally found abnormal liver biochemistries in the primary care setting.^54^ The sensitivity of abnormal aminotransferases in detecting NAFLD is poor since 55% to 79% of individuals with NAFLD may have normal transaminase levels.^21^ In patients with NAFLD, neither the degree of elevation nor the pattern of abnormal liver biochemistries are reliable in determining that the disease activity and risk of disease progression. In addition, changes of aminotransferase levels do not parallel changes in fibrosis stage preventing them from being a reliable surrogate for fibrosis progression.^55^

Mildly elevated serum ferritin is common in patients with NAFLD though it is typically not associated with increased hepatic iron stores.^56^ However, elevated serum ferritin and transferrin saturation in patients with suspected NAFLD should prompt genetic testing for hereditary hemochromatosis. A liver biopsy should be considered in a patient with suspected NAFLD who is homozygous or compound heterozygous for the C282Y mutation in the HFE gene to assess hepatic iron concentration and to evaluate for significant liver injury and fibrosis.

Mild elevations of autoantibodies are relatively common in patients with NAFLD. A similar phenomenon has been observed in liver disorders such as viral hepatitis and drug-induced liver injury and is generally considered as an epiphenomenon. A recent study from the NASH Clinical Research Network (NASH CRN), found that positive serum autoantibodies, defined as antinuclear antibody (ANA) titer ≥1:160 or antismooth muscle antibody (ASMA) ≥1:40, were present in 21% of patients with biopsy-proven NAFLD and they were not associated with more advanced histologic features.^57^ However, if there are additional features suggestive of autoimmune hepatitis, such as markedly elevated aminotransferase, high γ-globulin, or high serum immunoglobulin G, then a liver biopsy may be considered necessary to firmly establish the diagnosis.

Several models have been developed that combine laboratory testing, demographic variables, and clinical data to predict NASH with advanced fibrosis, but their detailed discussion is beyond the scope of this review article. The NAFLD fibrosis score (http://nafldscore.com/) is a promising bedside tool for identifying NAFLD patients who are at high risk for advanced fibrosis, and it employs six easily available variables (age, hyperglycemia, body mass index, platelet count, albumin, and AST/ALT ratio).^58^ In a recent meta-analysis of 13 published studies, the NAFLD fibrosis score had a pooled area under the curve of a receiver operating characteristic (AUROC) of 0.85 for predicting advanced fibrosis (stage 3 or 4). The NAFLD fibrosis score less than 1.455 had 90% sensitivity and 60% specificity to exclude advanced fibrosis, while a score greater than 0.676 had 67% sensitivity and 97% specificity to identify the presence of advanced fibrosis.^50^ Enhanced liver fibrosis (ELF) panel employs automated immunoassay of three serum markers of matrix constituents and mediators of matrix remodeling (hyaluronic acid, amino-terminal propeptide of type III collagen, and tissue inhibitor of matrix metalloproteinase 1).^59^ The ELF panel has an AUROC of 0.90 for detection of advanced fibrosis with a threshold of 0.3576 associated with a sensitivity of 80%, a specificity of 90%, a positive predictive value of 71%, and a negative predictive value of 94%.^60^

Studies suggest that hepatocyte apoptosis plays an important role in the pathogenesis of NASH. Hepatocyte apoptosis results in caspase 3 generated cleavage fragment of cytokeratin-18 (CK-18) being released. The serum CK-18 fragments can be measured by ELISA, and they significantly increased in patients with NASH compared with simple steatosis and normal controls.^61^ The circulating levels of CK-18 fragments have been shown to reflect disease activity, and change in their level may correlate with the change in NAFLD activity score.^37^ In a recent meta-analysis, the pooled AUROC of serum CK-18 for detection of NASH was 0.82 with a sensitivity and specificity of 78% and 87%, respectively.^50^

### Imaging

A number of imaging modalities have been employed in the diagnosis of NAFLD. Although several different imaging techniques are valuable in demonstrating steatosis, the ability of current imaging technologies to evaluate fibrosis and especially inflammatory activity is limited.

Abdominal ultrasound is a relatively inexpensive, noninvasive diagnostic test to demonstrate steatosis with excellent sensitivity in individuals with moderate to severe hepatic steatosis. Steatosis is visualized as increased echogenicity with bright liver echo pattern on ultrasound B-mode examination and increased attenuation.^62^ In a study of ultrasound paired with liver biopsy the reported sensitivity ultrasound for the detection steatosis of 64% to 91%, and a specificity of 93% to 97%.^63^,^64^ Morbid obesity was associated with a lower sensitivity, and higher degrees of steatosis with a greater sensitivity.

Noncontrast abdominal CT is also useful in demonstrating hepatic steatosis. When contrast enhanced CT is performed, the portal phase images should be employed for the determination of steatosis though contrast may result in decreased sensitivity and specificity compared with noncontrast scans.^65^ On a CT scan, hepatic steatosis is visualized as decreased attenuation of the liver, resulting in the liver appearing darker than the spleen and is associated with decreased liver attenuation index, which is the difference between the mean hepatic and splenic attenuation in Hounsfield units. The sensitivity of a CT scan for detecting steatosis greater than 30% was as high as 82% in a study involving potential living liver donors.^65^

Abdominal magnetic resonance imaging (MRI) is a sensitive technique for demonstrating steatosis. T1-weighted gradient-echo magnetic resonance images are acquired with an echo time such that water and lipid spins are in phase or opposed phase allowing lipid quantitation by relative loss of signal intensity on opposed-phase images compared with that on in-phase images.^66^ In a study of potential living donors MRI demonstrated a sensitivity, specificity, and accuracy of 100%, and 92.3%, and 93%, respectively, for detection of steatosis in patients with greater than 20% steatosis by liver biopsy.^67^ Magnetic resonance spectroscopy has been employed to quantitate hepatic steatosis by measuring hepatic triglyceride content although its use remains primarily investigational.^68^

Heterogeneity of hepatic steatosis including focal sparing is a relatively common finding with each of the imaging modalities.^69^–^74^ The most common locations are the gallbladder fossa, and the areas adjacent to the porta hepatis and falciform ligament. Conversely, focal fatty infiltration can also be observed where steatosis is increased in only one region of the liver.

Transient elastography is an ultrasound-based noninvasive method of assessing of fibrosis through measurement of liver stiffness. Although insensitive for detection of early fibrosis, transient elastography can be useful in screening for advanced fibrosis in many patients. In a study involving patients with NAFLD, transient elastography demonstrated a sensitivity of 91% and a specificity of 75% for detecting stage 3 or higher fibrosis with positive and negative predictive values of 52% and 97%, respectively.^75^ Unfortunately, body habitus can limit the application of this study since there is an increased failure rate in obtaining a successful transient elastography measurement with increasing degree of obesity. Also, transient elastography is not currently commercially available in the United States. Magnetic resonance elastography is a promising technique that measures liver stiffness over a larger region of the liver than transient elastography; this research tool is currently available at a limited number of centers.

### Liver biopsy

Liver biopsy remains the gold standard for characterizing the histology of NAFLD. It can play an important role in the diagnosis of NAFLD, but it is expensive and carries risk of morbidity and very rarely mortality. Histological examination of liver tissue allows for exclusion of competing etiologies as well as for the assessment of coexisting liver diseases. Currently, liver biopsy is the only tool available for assessing the degree of inflammation and cell injury and to stage for the degree of fibrosis. It is invaluable to differentiate simple steatosis from NASH.

**Figure 1:**
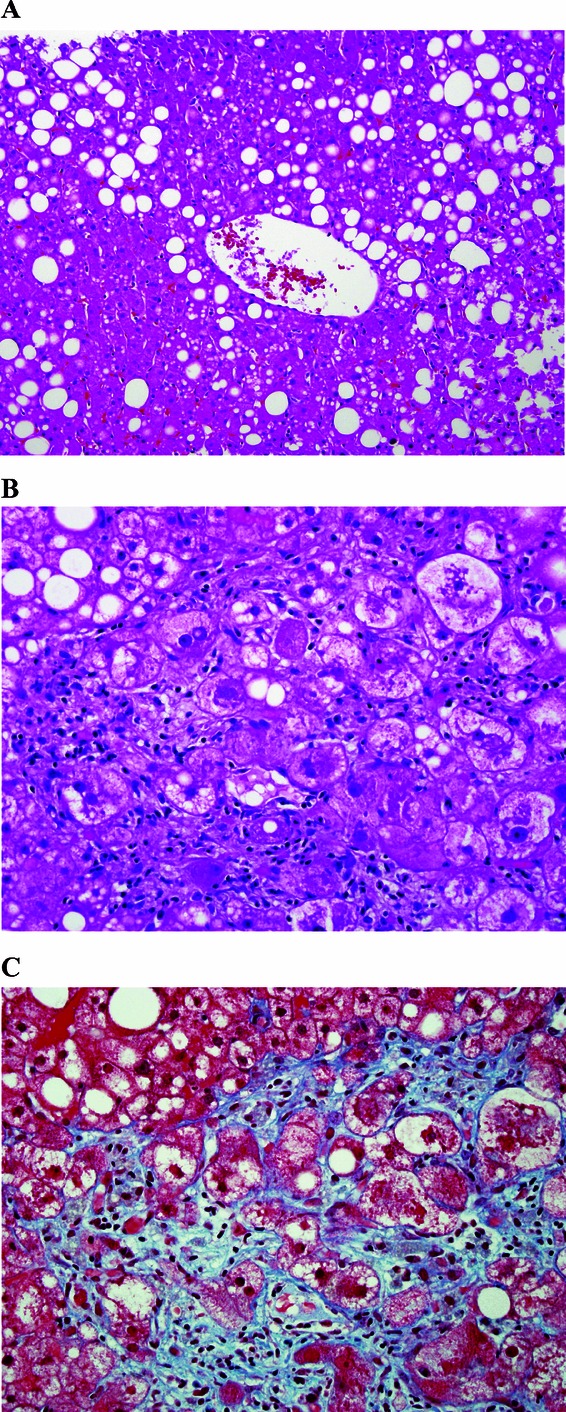
(A) Liver histology demonstrating moderate macrovesicular steatosis around the central vein. Hematoxylin and eosin staining, with magnification of 200×. (B) Liver histology demonstrating active steatohepatitis with steatosis, ballooned hepatocytes, inflammatory infiltrate, and Mallory's Hyaline. Hematoxylin and eosin staining, with magnification of 400×. (C) Liver histology demonstrating steatohepatitis with extensive pericellular fibrosis. Trichrome staining, with magnification 400×. Figure courtesy of David Kleiner, MD, National Cancer Institute.

Macrovesicular steatosis is a predominant feature of NAFLD, and the presence of steatosis in greater than 5% of hepatocytes is generally accepted as fatty liver (Fig. 1A).^76^ In addition to steatosis, common histologic findings in NASH include hepatocyte ballooning, lobular inflammation that is either mixed type or neutrophil predominant, and varying degrees of fibrosis (Figs. 1B and C).^77^ There are no histologic features that reliably differentiate NASH for alcoholic hepatitis, and the term *NASH* was originally employed in a report from the Mayo Clinic regarding 20 patients with a liver disease that histologically mimicked alcoholic hepatitis in patients without significant alcohol intake.^78^ A histopathological classification system for NASH was originally developed by Brunt *et al.*, with histologic features distinctive to NASH employed in determining the necroinflammatory activity (grade) and architectural alterations (stage).^79^ The NAFLD activity score (NAS), an unweighted sum of steatosis, inflammation, and ballooning scores, was developed by the NASH CRN as a tool to quantify changes in liver histology in NAFLD therapeutic trials.^76^ There is not a threshold value of NAS that reliably identifies the presence of NASH.^80^

There can be significant sampling error due to the small portion of the liver sampled by a biopsy and the inhomogeneous distribution of the histologic lesions of NASH. This sample variability is moderate for hepatocyte ballooning and perisinusoidal fibrosis and is somewhat higher for lobular inflammation.^81^,^82^

## Pathogenesis of NAFLD

Several pathophysiologic mechanisms have been proposed to explain the basis for fat accumulation, liver injury, and fibrosis in NAFLD, but their detailed discussion is beyond the scope of this review article. Interested readers are alerted to several recent comprehensive reviews on this subject.^7^,^8^,^10^,^83^,^84^ Despite considerable research in this area, the pathogenesis of NAFLD remains incompletely understood. It has been very challenging to differentiate causative factors from associated phenomena and downstream effects. Although there is an increased understanding of the pathogenesis of hepatic fat accumulation, there are critical knowledge gaps in our understanding of mediators and mechanisms of hepatocyte injury, mediators of stellate cell activation, and fibrosis. It remains a puzzle why some individuals with NAFLD have advanced histological features and develop cirrhosis whereas others with comparable risk factor profile have simple steatosis with minimal or no disease progression. A genetic basis for inter-individual phenotypic variability is strongly suspected, but genetic studies are very limited in individuals with histologically characterized NASH.

Insulin resistance is nearly universal in NAFLD and it is believed to play a crucial role in the pathogenesis of NAFLD. In adipocytes, insulin resistance results in increased activity of hormone-sensitive lipase, which results in lipolysis of triglycerides and release of free fatty acids into circulation. Fatty acids taken up by hepatocytes from circulation and produced by *de novo* lipogenesis undergo esterification resulting in hepatocytes steatosis. Initially it was proposed that NASH resulted from a “two-hit” mechanism with hepatocyte steatosis being the initial metabolic insult that then allows a second injury leading to NASH.^85^ However, subsequent research has cast significant doubt on this paradigm, and it is now widely believed that free fatty acids and their metabolic products (e.g., diacylglycerol) and sequelae (e.g., free radicals) are the likely mediators of hepatocyte injury.^8^,^86^–^89^

At a cellular level, several different mechanisms have been proposed for causing hepatocyte injury, including apoptosis, perturbations in autophagy, mitochondrial dysfunction, alterations in natural killer T cell and Kupffer cell function, and an increase in inflammatory cytokines.^8^,^90^–^96^ Apoptosis appears to play an important role in hepatocyte death in NAFLD, and free fatty acids could be the primary mediators of hepatocyte apoptosis (lipoapoptosis).^83^,^90^ In addition, phagocytosis of hepatocyte apoptotic bodies by stellate cells leads to their activation and likely plays an important role in fibrosis.

## Treatment of NALFD

### Lifestyle modification

Lifestyle modification is the cornerstone of treatment of NAFLD. These interventions are not only effective in improving NAFLD but also associated conditions such as metabolic syndrome, T2DM, and the related risk of cardiovascular disease.

Weight reduction plays an important role in the treatment of NASH. Weight loss has been shown to decrease hepatic steatosis and improve abnormal aminotransferase levels.^97^–^99^ Weight loss can be an effective treatment to improve the histology of NASH if patients can attain sufficient weight reduction. In a study by Harrison *et al.*, subjects with biopsy-proven NASH who lost 5% of body weight had improvement in insulin sensitivity and hepatic steatosis compared with those who lost less than 5% of their body weight.^100^ However, it was only in subjects who achieved at least 9% weight reduction that there was significant improvement in inflammation, ballooning, and NAS. A randomized controlled trial (RCT) involving patients with biopsy-proven NASH by Promrat *et al.* examined the efficacy of lifestyle intervention using a combination of diet, exercise, and behavior modification compared to the control group that received structured education.^101^ The primary end-point in this study was improvement in liver histology. Participants who achieved the study weight loss goal of at least 7% had significant improvements in steatosis, lobular inflammation, and ballooning injury. Percent weight reduction correlated significantly with improvement in NAS. Weight loss has been shown to prevent progression of fibrosis in NASH.^37^

However, very rapid weight loss may lead to increased portal inflammation and fibrosis. In a small study of severely obese patients with NAFLD who were placed on a very low calorie formula diet resulting in a median weight loss of 34 kg over an 8-week period, 24% of patients developed mild portal inflammation or portal fibrosis.^102^ Therefore, one should be cautious in recommending very low calorie diets for individuals with NAFLD.

### Weight reduction surgery

Because NAFLD is present in the majority of patients who undergo bariatric surgery, there has been an interest in foregut bariatric surgery as a potential treatment option for NASH. Currently, there are no RCTs that have examined foregut bariatric surgery as a treatment option for NAFLD or NASH. However, several retrospective and prospective cohort studies have compared liver histology in the severely obese individuals before and after bariatric surgery. Unfortunately, a majority of them do not have uniform histologic evaluation by post-bypass liver biopsies and instead performed biopsies at varying intervals and only in selected patients undergoing other surgical procedures such as abdominal hernia repair. However, one exception is the seminal study by Mathurin *et al.*, that prospectively correlated clinical and metabolic data with liver histology before and 1 and 5 years after bariatric surgery in 381 adult patients with severe obesity.^103^ There was a significant improvement in steatosis and ballooning at 1 and 5 years following bariatric surgery compared to baseline. In patients with probable or definite NASH at baseline, there was significant improvement in steatosis, ballooning, and NAS and resolution of probable or definite NASH at 1 and 5 years following bariatric surgery. The majority of histological benefits were present at 1 year with no differences in liver histology between 1 and 5 years following bariatric surgery. Because no patient in this study had cirrhosis at baseline, the effect of bariatric surgery in patients with cirrhosis could not be evaluated.

There are two meta-analyses that evaluated the influence of bariatric surgery on liver histology in adults with NAFLD. Mummadi *et al.* found that steatosis, steatohepatitis, and fibrosis improve or completely resolve after bariatric surgery in a significant proportion of patients.^104^ However, a Cochrane review concluded that lack of RCTs or other high-quality clinical studies prevents definitive determination of benefits and risks of bariatric surgery as a treatment option for patients with NASH.^105^

A recently published multi-society practice guideline concluded that it is premature to consider foregut bariatric surgery as an established option to specifically treat NASH.^15^ However, it concluded that foregut bariatric surgery is not contraindicated in otherwise eligible obese individuals with NAFLD or NASH.

### Vitamin E

Oxidative stress has been proposed as an important mediator of hepatic injury in NASH.^88^,^106^,^107^ Vitamin E comprises a series of closely related compounds with antioxidant activity that have been employed in several therapeutic trials of NASH, although small sample sizes, differences in vitamin E preparations, and differences in endpoints have made them difficult to compare. The PIVENS trial, a recent randomized double blind placebo controlled trial, is the largest study to investigate the effectiveness of vitamin E supplementation on nondiabetic adults with histologically confirmed NASH.^108^ This study employed unmodified RRR-alpha-tocopherol administered as a once daily dose of 800 IU given for 96 weeks. Vitamin E supplementation resulted in significant improvement in pathologic features of NASH with improvement in NAS seen in 42% of patients receiving vitamin E compared with 19% of patients receiving placebo with a number needed to treat of 4.4. Compared with placebo, vitamin E significantly improved aminotransferases as well. Vitamin E was well tolerated in this trial. The effectiveness of vitamin E supplementation has not been evaluated in diabetic patients with NASH or in patients with NASH-related cirrhosis.

Some concerns have been raised regarding the long-term safety of vitamin E, although current data suggest that serious toxicity from vitamin E is likely very small if present. A recent RCT of vitamin E administered at a dose of 400 IU/day found a statistically non-significant increase in prostate cancer risk in the vitamin E group with an absolute increased risk of 1.6 per 1,000 person years of vitamin E use.^109^ Whether vitamin E supplementation may increase all-cause mortality remains controversial. While some early meta-analyses suggested a possible increase in all-cause mortality associated with vitamin E supplementation, subsequent studies have failed to demonstrate any increased mortality.^110^–^115^

### Insulin-sensitizing agents

Insulin-sensitizing agents have been investigated extensively in therapeutic trials since insulin resistance is believed to play an important role in the pathogenesis of NAFLD.

Metformin has been employed in a number of therapeutic trials of NASH. While several small open-label studies suggested some improvement in aminotransferase levels with metformin therapy, a study in which only the metformin arm underwent biopsy suggested that it might lead to histologic improvement.^116^ However, subsequent randomized, placebo controlled clinical trials have failed to show a significant difference in liver histology in nondiabetic patients with insulin resistance and NASH.^117^,^118^ Because metformin does not have a significant effect on liver histology in patients with NASH compared with lifestyle modification alone, the use of metformin as therapy for NASH is not recommended.^15^

Thiazolidinediones (TZDs) are oral antidiabetic medications that increase insulin sensitivity by activation of peroxisome proliferator-activated receptors present in a number of tissues, including liver, skeletal muscle, and adipose tissue. An early nonrandomized trial, involving 22 patients with biopsy-proven NASH (including 50% with impaired glucose tolerance and diabetes) treated with rosiglitazone 4 mg twice daily for 48 weeks, demonstrated improvement in inflammation, hepatocyte ballooning, and fibrosis on an end-of-treatment liver biopsy.^119^ However, weight gain occurred in 67% of patients with a median body weight increase of 7.3%.

The RCTs of TZDs have generally shown histologic improvement with TZDs in patients with NASH, although there has been variability in certain parameters, such as improvement in inflammation and fibrosis. In an early double-blind placebo controlled study by Belfort *et al.*, pioglitazone (45 mg/day) combined with a hypocaloric diet significantly improved steatosis, hepatocyte ballooning, and inflammation compared with a hypocaloric diet alone in patients with biopsy-proven NASH and either T2DM or insulin resistance.^120^ Improvement in the NAS was seen in 73% of patients treated with pioglitazone compared to 24% of placebo-treated patients (*P* < 0.001), and there was a trend toward improvement in fibrosis in patients receiving pioglitazone (*P* < 0.08). A randomized trial of 63 patients with biopsy-proven NASH by Ratziu *et al.*, found that rosiglitazone treatment (4 mg/day for the first month and 8 mg/day thereafter) for one year improved aminotransferases and hepatic steatosis, but not necroinflammation, fibrosis or NAS.^121^ A two-year open-label extension phase of this study demonstrated similar results with no significant improvement in hepatocyte ballooning, intralobular inflammation, fibrosis, or NAS seen in the rosiglitazone treatment group compared with the control group.^122^ Aithal *et al.*, conducted a randomized, placebo-controlled trial of lifestyle intervention with either pioglitazone (30 mg/day) or placebo for 12 months in a total of 74 nondiabetic patients with NASH.^123^ Although steatosis did not improve significantly compared to placebo, cellular injury and fibrosis improved significantly. Weight gain was observed in the TZD group in each of these studies and ranged from 1.5 to 2.77 kg, whereas the placebo control groups lost 0.55 to 1 kg.

The PIVENS study is a recent large RCT that randomized 247 nondiabetic patients with biopsy-proven NASH to pioglitazone (30 mg/day), vitamin E (800 IU/day), or placebo for 24 months.^124^ The primary endpoint was an improvement in NAS by at least 2 points, with at least a one-point improvement in hepatocellular ballooning and a one-point improvement in either the lobular inflammation or steatosis score, and no increase in the fibrosis score.^108^ This endpoint was achieved in 34% of the pioglitazone group (*P* = 0.04 vs. placebo) and 43% of the vitamin E group (*P* = 0.001 vs. placebo) compared with 19% in the placebo group. The resolution of NASH, a key secondary end point, was achieved in significantly higher percentage of patients receiving pioglitazone compared with placebo (47% vs. 21%, *P* = 0.001). Similar to prior trials, pioglitazone was associated with a 4.7 kg weight gain compared to placebo. A recent meta-analysis that included four high quality randomized placebo controlled trials showed that TZDs significantly improved steatosis (OR 3.39, 95% 2.19–5.25), inflammation (OR 2.58, 95% CI: 1.68–3.97), and ballooning (OR 2.11, 95% CI:1.33–3.36), but not fibrosis (combined OR 1.40, 95% CI 0.87–2.24).^125^ When studies involving pioglitazone alone were analyzed, there was statistically significant improvement in fibrosis (combined OR 1.68, 95% CI: 1.02–2.77).

The addition of metformin to rosiglitazone has recently been investigated but metformin did not offer additional histologic improvement over rosiglitazone treatment alone in two open-labeled RCTs and more importantly metformin did not mitigate TZD associated weight gain.^118^,^126^

There is considerable debate about the long-term safety of TZDs with reference to increased risk of cardiovascular events, congestive heart failure (CHF), bladder cancer, and bone loss. A meta-analysis of trials involving rosiglitazone demonstrated a significant increase in the rate of myocardial infarction (OR 1.43, 95% CI 1.03 to 1.98, *P* = 0.03).^127^ This is distinct from the meta-analysis of 19 trials of pioglitazone enrolling a total of 16,390 patients with T2DM, pioglitazone treatment was associated with a significant reduction in the primary outcome of death, myocardial infarction, or stroke (*P* = 0.005).^128^ However, there was increased incidence of CHF with pioglitazone (2.3% vs. 1.8% in the control group, *P* = 0.002). Therefore, caution must be exercised when considering TZDs in patients with preexisting cardiac disease. Owing to increased risk of cardiac events, rosiglitazone availability is highly restricted in the United States and is no longer marketed in Europe.

### Statins, omega-3 fatty acids, and ursodeoxycholic acid

Dyslipidemia is almost universal in patients with NAFLD, and the effect of lipid-lowering therapy with statins on NALFD has been evaluated in several studies. The St. Francis Heart Study demonstrated that atorvastatin 20 mg combined with vitamins C and E is effective in reducing the odds of having hepatic steatosis as determined by CT although no histologic evaluation was performed.^129^ The *post hoc* analysis of the Greek Atorvastatin and Coronary Heart Disease Evaluation (GREACE) study of 227 patients with possible NAFLD based on moderately abnormal liver tests at baseline treated with a statin demonstrated improvement in liver tests without an increase in liver-related adverse effects compared with controls.^130^ Several other studies have demonstrated that statins are safe in patients with liver disease, and there is no evidence that patients with NAFLD are at increased risk for serious liver injury from statins compared with those without liver disease.^131^–^134^ There are no RCTs with histological end points that have examined the use of statins in the treatment of NASH, and thus statins specifically to treat NASH cannot be advocated at this time.^15^

Treatment of NAFLD with fish oil supplementation or polyunsaturated fatty acids that are enriched in fish oil omega 3 fatty acids has been investigated in animal studies and a small number of preliminary human studies. Epidemiologic studies have suggested that there may be an inverse relationship between the level of fish oil intake and risk of NAFLD.^135^,^136^ However, the association is relatively modest and was not statistically significant in some studies after adjusting for confounding factors. Several small nonrandomized open-label study of omega-3 fatty acids alone or with supplements such as olive oil have found improvement in liver tests, serum triglycerides, and steatosis by ultrasound.^137^–^140^ A large multicenter trial of the omega-3 fatty acid eicosapentanoic acid to treat NASH is ongoing in the United States. At this point, it is premature to recommend omega-3 fatty acids for the treatment of NAFLD although they may be considered for the treatment of hypertriglyceridemia.

There has been interest in the use of ursodesoxycholic acid (UDCA) to treat NAFLD although studies to date have yielded disappointing results. UDCA is a secondary bile acid that is approved for the treatment of primary biliary cirrhosis and has effects on cholesterol absorption and inflammation. Initial small uncontrolled clinical studies suggested that UDCA may offer benefit to individuals with NASH. However, a two-year prospective, double-blind trial of UDCA (13–15 mg/kg per day) of 166 patients failed to demonstrate improvement in laboratory data or liver histology.^141^ Subsequent studies have employed high dose UDCA with mixed results. A RCT of high-dose UDCA (28–35 mg/kg per day) given for 12 months in patients with NASH by Ratziu *et al.*, demonstrated that UDCA improved transaminase levels and markers of insulin resistance and fibrosis.^142^ Critically, no histologic evaluation was performed. Leuschner *et al.* performed a double-blind, randomized, placebo-controlled trial of high-dose UDCA (23–28 mg/kg/day) in 185 patients with histologically proven NASH.^143^ The treatment was provided over 18 months with both pre- and posttreatment liver biopsies. Although lobular inflammation was improved in patients in the treatment group, there was no improvement in fibrosis and no significant difference in NAS between the treatment and control group. In summary, there is no evidence that UDCA is effective to treat NASH.

### Emerging therapies

Table 4 describes selected compounds that are being tested in large phase 2/3 studies. A large randomized placebo controlled trial of two doses of eicosapentanoic acid is near completion in the United States and its results are eagerly awaited. Pentoxifylline has shown encouraging histological benefits in several small studies and it is a suitable candidate for further testing in large multicenter RCTs. The NASH CRN is conducting a multicenter RCT of obeticholic acid in adults with NASH and a multicenter RCT of cysteamine bitartrate in children with NASH. Their results will not be available until 2014 and 2015, respectively. Obeticholic acid is a novel FXR agonist, whereas cysteamine bitartrate is a potent antioxidant.

**Table 4:** Selected compounds with high therapeutic potential that are currently being investigated in phase2/3 studies

Compound	Nature of the trial	Potential mechanism of action	Primary end point	Comment
Eicosapentanoic acid	Multicenter phase 2/3 study in the United States; sponsored by Mochida Pharmaceuticals	Decreased lipogenesis and improved insulin sensitivity	Liver histology	To be completed soon; results awaited
Pentoxifylline	Several small studies have shown histological benefits	Anti-TNF-α	Liver histology	Suitable agent for large-scale definitive studies
Obeticholic acid	Large placebo-controlled, phase 2b is under way in the United States; conducted by the NASH CRN under a CRADA agreement with Intercept Pharmaceuticals	Farsenoid X receptor agonist	Liver histology	Results will become available in 2014
Cysteamine bitartrate	Large placebo-controlled, phase 2b trial in children with NASH is under way; conducted by the NASH CRN under a CRADA agreement with Raptor Pharmaceuticals	Potent antioxidant	Liver histology	Results to become available in 2015
GFT 505	Multicenter, placebo-controlled RCT to be initiated soon; sponsored by Genfit	GFT 505 is a dual PPAR α/δ agonist	Liver histology	To be initiated soon
GS 6624	Two separate phase 2b studies to be initiated internationally by Gilead Pharmaceuticals	GS 6624 is a parenteral compound, and it is a monoclonal antibody against a lysyl oxidase–like molecule	Reversal of cirrhosis by histology is the primary end point for the cirrhosis study; however, progression of fibrosis is the end point for the advanced fibrosis study	To be initiated soon
	In adults with compensated cirrhosis			
	In adults with advanced fibrosis			

## Surveillance for the development of complications

In addition to their risk of developing cirrhosis, liver failure, and hepatocellular cancer, patients with NAFLD are at significantly higher risk for developing diabetes and cardiovascular disease, and thus there should be heightened attention to monitoring for the development of these conditions.

In patients with NASH related cirrhosis, regular surveillance for cirrhosis related complications such as hepatocellular carcinoma (HCC) and esophageal varices should be performed. As with other causes of cirrhosis, surveillance for HCC should be performed every 6 months with abdominal ultrasound, or intravenous contrast enhanced abdominal CT or MRI. Yearly cumulative incidence of HCC in patients with NASH-related cirrhosis was found to be 2.6% in one series. This was less than the yearly cumulative incidence in patients with hepatitis C cirrhosis of 4.0% though the difference was not statistically significant.^144^

## Conclusions

Individuals with obesity and T2DM are at significantly higher risk for NAFLD. The incidence of NAFLD is rapidly increasing throughout the world due to the increasing frequency of obesity and T2DM. The NAFLD is a spectrum of chronic liver diseases ranging from simple steatosis, which is relatively benign from a liver standpoint, to NASH, which can progress to cirrhosis and liver failure. The diagnosis of NAFLD requires imaging evidence of hepatic steatosis while excluding competing etiologies, such as significant alcohol consumption, viral hepatitis, and hemochromatosis. Liver biopsy remains the gold standard for diagnosing NASH and for assessing fibrosis. Recent advances in laboratory testing and noninvasive imaging have shown promise for identifying steatohepatitis and advanced fibrosis in individuals with NAFLD. Weight loss of at least 5% is required to improve steatosis, whereas weight loss in the range of 7–10% may be needed to improve steatohepatitis. A number of pharmacologic therapies have been evaluated in NASH, and agents such as vitamin E and TZDs have shown some promise. Ongoing studies hold promise for developing more effective diagnostic tests and therapies.
